# Maxillary Sinus Lift Using Autologous Periosteal Micrografts: A New Regenerative Approach and a Case Report of a 3-Year Follow-Up

**DOI:** 10.1155/2018/3023096

**Published:** 2018-07-24

**Authors:** Saturnino Marco Lupi, Arianna Rodriguez y Baena, Claudia Todaro, Gabriele Ceccarelli, Ruggero Rodriguez y Baena

**Affiliations:** ^1^Department of Clinical Surgical, Diagnostic and Pediatric Sciences, University of Pavia, Pavia, Italy; ^2^Department of Public Health, Experimental Medicine and Forensic, Human Anatomy Unit, University of Pavia, Pavia, Italy

## Abstract

This case report discusses about an innovative bone regeneration method that involves the use of autologous periosteal micrografts, which were used for a maxillary sinus floor lift in a 52-year-old female patient. This method allows for harvesting of a graft that is to be seeded on a PLGA scaffold and involves collection of a very little amount of palatal periosteal tissue in the same surgical site after elevation of a flap and disaggregation of it by using a Rigenera® filter. Histological samples collected at the time of implant installation demonstrate a good degree of bone regeneration. The clinical and radiographic outcomes at the 3-year follow-up visit showed an adequate stability of hard and soft tissues around the implants. This report demonstrates the possibility to obtain a sufficient quality and quantity of bone with a progenitor cell-based micrograft and in turn make the site appropriate for an implant-supported rehabilitation procedure, with stable results over a period of two years.

## 1. Introduction

Tooth loss causes alveolar bone resorption that often limits implant placement. In the superior maxilla, this process is associated with the pneumatization of the sinus [1–3].

Since the 60s, numerous surgical techniques have been proposed for the regeneration of maxillary bone defects. When the residual bone height is inadequate for implant placement, in case of favorable prosthetical spaces, a sinus lift is considered a safe procedure with predictable results [4–10]. Implant placement is contraindicated if the residual bone height is less than 5 mm [11].

Current treatment options for bone defects include autologous, homologous, xenologous, and allogenous grafts; artificial bone substitutes can be synthetic or bioceramic cements or a blend of two or more materials [12]. Although several studies have been conducted to identify the best graft material for sinus floor augmentation, a final consensus has not been reached [4]. Autologous bone grafts represent the gold standard graft material because these exhibit osteoinductive, osteoconductive, and osteogenic properties. However, the use of this material remains limited owing to rapid resorption, collection of inadequate amounts of tissue if harvested intraorally, donor site morbidity, and high biologic cost [13]. Alternatively, the alloplastic bone substitutes and the xenologous bone show high availability, biocompatibility, and good mechanical support and also have adequate porosity that allows for penetration of blood capillaries, which is essential for the supply of oxygen, nutrients, and growth factors [14–19]. However, these bone substitutes are limited by the fact that they do not carry osteogenic cells and osteoinductive molecules, which are important for tissue regeneration [20–23].

In the last twenty years, researchers have shown renewed interest in developing new regeneration methods. Researchers are particularly focusing on mesenchymal stem cells because they represent a self-renewable reservoir of cells that can proliferate and differentiate at the same time. Thus, if correctly transplanted, mesenchymal stem cells are able to regenerate a particular tissue [24–28].

Unfortunately, stem cell therapies require both highly developed technologies and methods, which are not yet allowed to be used in many countries (e.g., laboratory handling of stem cells to produce tissues). Moreover, a few researchers have reported that stem cell therapies could increase the risk for tumor growth [29–34].

Recently, a class I medical device (Rigeneracons®, Human Brain Wave S.R.L., Torino, Italy) has been introduced in clinical practice in order to disaggregate a portion of tissue and obtain 50 *μ*m viable micrografts full of progenitor cells, while maintaining their regenerative and differentiation potentials. These micrografts can be obtained from a sample of autologous connective tissue few millimeters in length, which is harvested directly during the surgery, even from the same surgical site, and can be immediately used without any handling or cell culture [35].

The aim of this report is to present a clinical case in which autologous micrografts with a high percentage of progenitor cells were seeded on a PLGA hydroxyapatite- (HA-) enriched scaffold for a sinus floor lift augmentation procedure and to present the histological features shown by the sample collected at the implant site and the radiographic aspect obtained three years after the lift procedure.

## 2. Case Report

### 2.1. Materials and Methods

The procedure discussed in this case report was performed at the Department of Clinical Surgical, Diagnostic and Paediatric Sciences, University of Pavia, Italy, and the procedure was approved by the University Ethics Committee (recorded March 2014).

**Figure 1:**
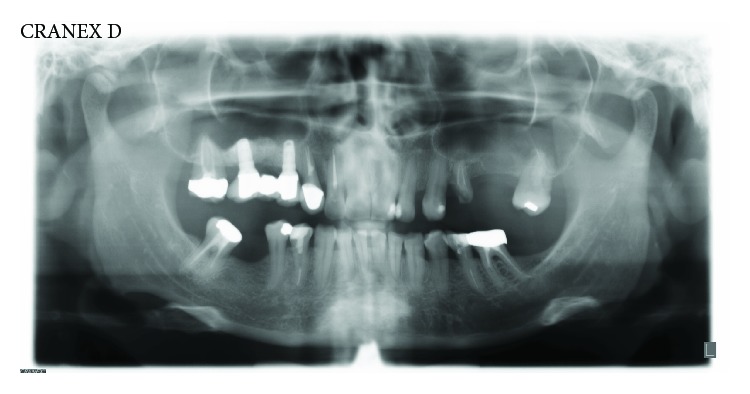
Preoperative panoramic radiograph of the patient.

**Figure 2:**
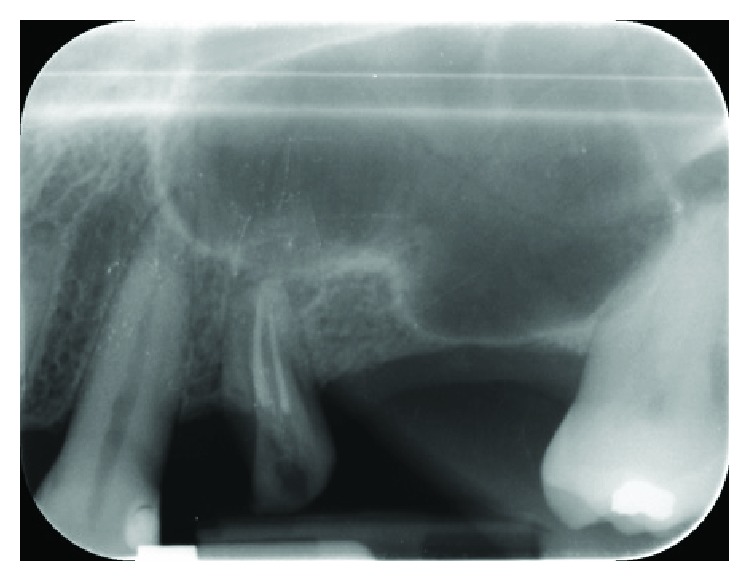
Intraoral radiograph (Rinn® collimator) of the surgery site.

A 52-year-old woman, with a good health status (ASA score: 0), was enrolled for the study; written informed consent was obtained from the patient to have the case details and any accompanying images anonymously published. She was indicated for a prosthetic implant rehabilitation procedure in the second quadrant after a maxillary sinus lift procedure for atrophy of the maxillary bone at the bicuspid and molar level (1 mm residual bone crest height) in order to collect enough bone to install two endosseous implants (Figures 1 and 2).

The patient was prepared for the surgery with scaling and root planning two weeks prior to the sinus floor lift. The surgery was performed under antibiotic prophylaxis: amoxicillin plus clavulanic acid (Augmentin, GlaxoSmithKline S.p.A., Verona, Italy), 2 gr 1 hour before the surgery. For the local anesthesia, articain 4% with 1/200000 epinephrine was used.

**Figure 3:**
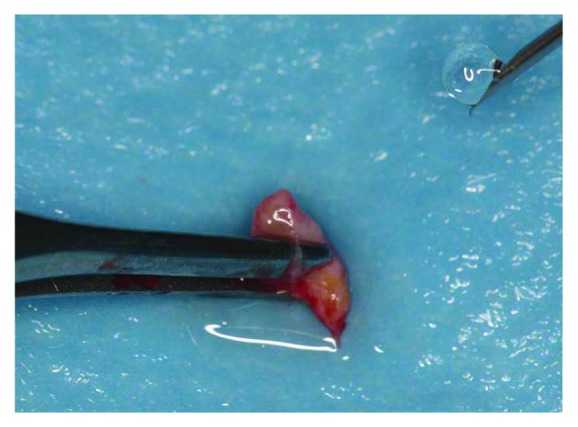
Connective tissue collected directly from the surgery site.

**Figure 4:**
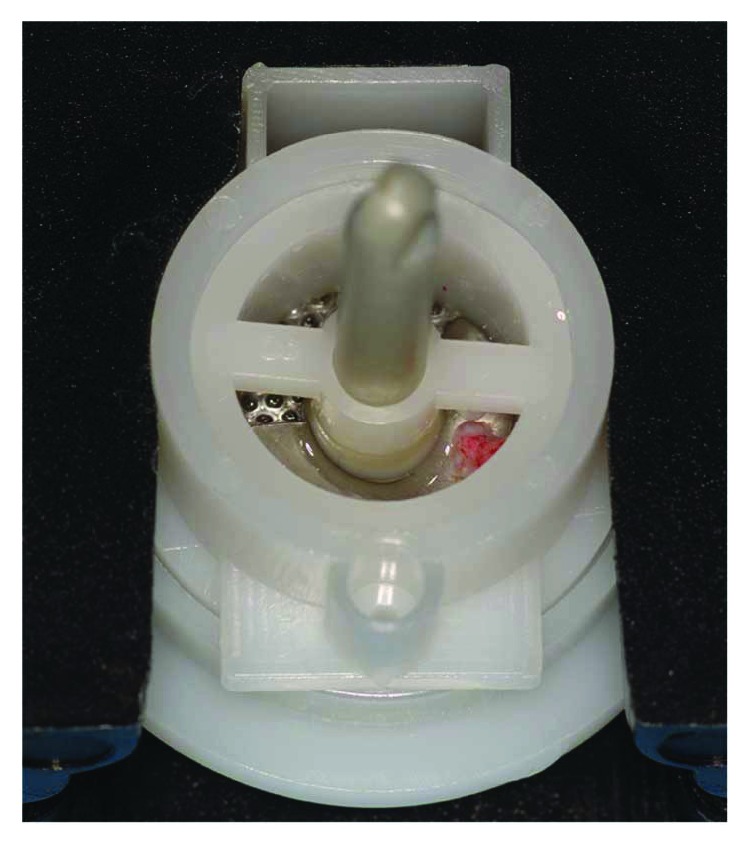
Tissue graft disaggregation with the Rigeneracons device, according to the manufacturer's instructions: 1 ml of sterile saline solution, performed for 120 seconds with implant contra-angle at 15 NCm and 70 rpm.

A full-thickness flap was lifted via mesial and distal relief incisions. From the palatal flap, a 3 mm periosteal sample was harvested and then washed with a sterile saline solution. Then, it was inserted in the Rigeneracons filter with 1 ml of sterile saline for the disaggregation process (Figures 3 and 4).

**Figure 5:**
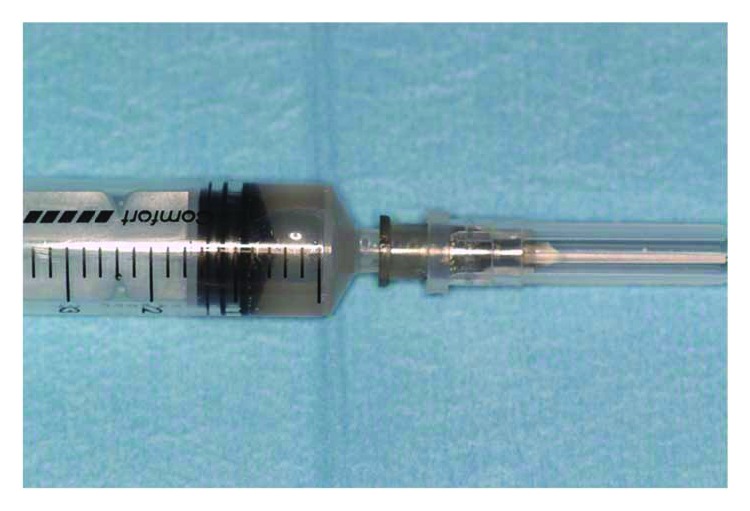
The syringe with progenitor cell-enriched suspension obtained via the periosteum disaggregation process.

**Figure 6:**
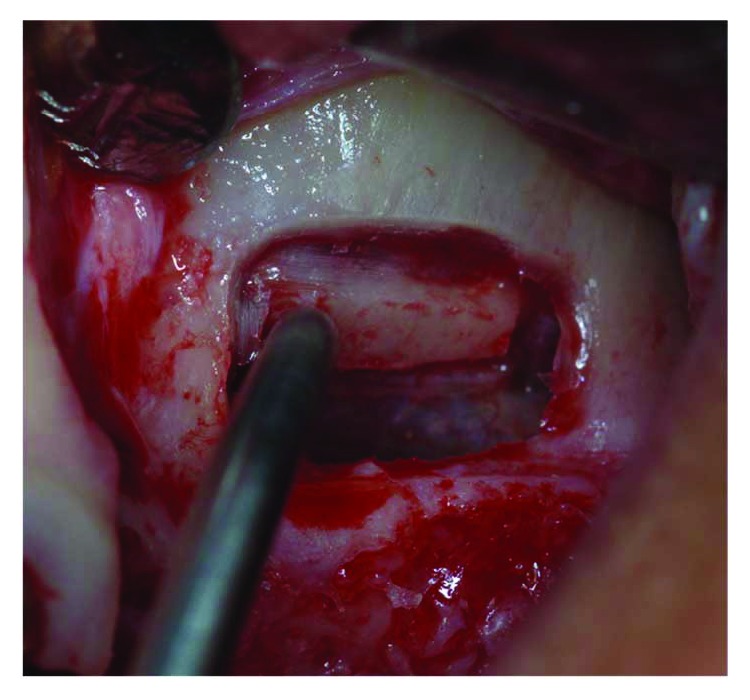
The window elevation.

**Figure 7:**
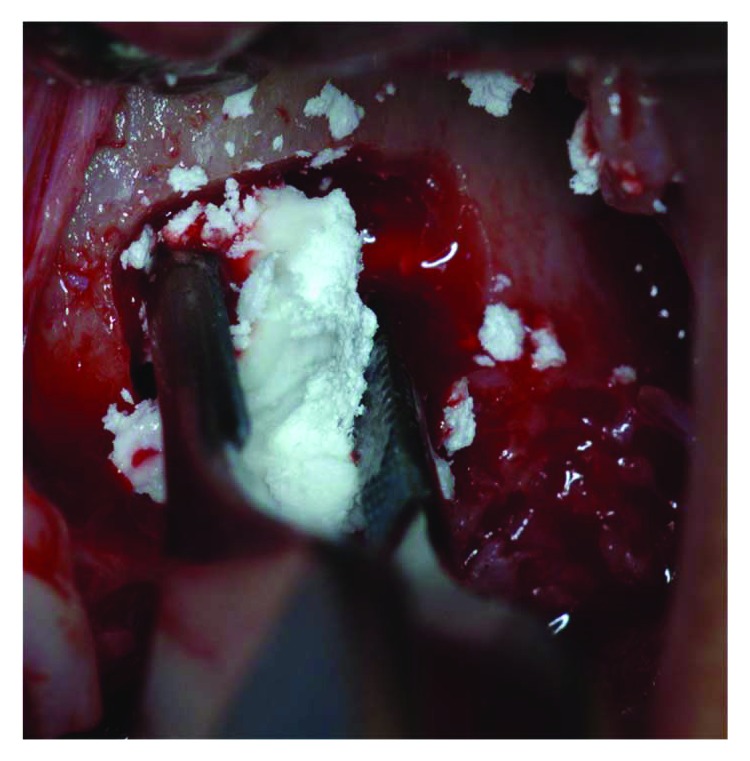
The biocomplex graft placement in the maxillary sinus, under the Schneider membrane.

**Figure 8:**
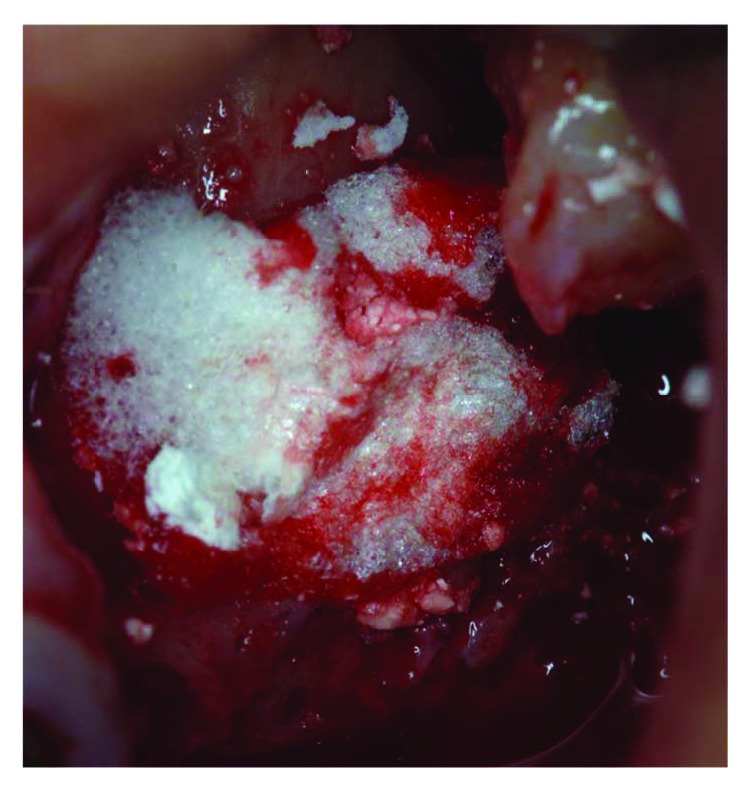
Covering the osteotomy access with collagen and resorbable membrane.

Tissue graft disaggregation was performed for 2 minutes at 70 rpm and 15 Ncm torque, and the cell suspension was withdrawn with a sterile syringe and added to the PLGA-HA scaffold (Alos®, Allmed srl, Lissone, MB, Italy) in order to be grafted into the new subantral cavity (Figure 5). In the meantime, the receiving site was prepared according to the standard protocol used for lateral sinus floor augmentation (Figure 6) [11, 14]. The wall osteotomy was performed with Piezosurgery® (Mectron S.p.A., Carasco, GE, Italy) using an OT5 insert. A resorbable membrane (Bio-Gide®, Geistlich Pharma AG, Wolhusen, Switzerland) was positioned in the newly formed subantral cavity to preserve the sinus membrane, and the space was then filled with the blend of PLGA and micrograft (Figures 6 and 7). The bone window was covered with collagen sponges (Gingistat®, Pierre Rolland Pharmaceutical, Merignac, Aquitaine, France) soaked in the cell suspension and a resorbable membrane (Bio-Gide, Geistlich Pharma, Wolhusen, Switzerland) (Figure 8). The flap was sutured with a 4-0 PTFE suture (Omnia S.p.A, Fidenza, PR, Italy).

During the postoperative period, the patient received antibiotic therapy (1 gr every 12 hours of amoxicillin + clavulanic acid for 7 days) and performed oral rinses with chlorhexidine 0.2% (Curasept®, Curaden Healthcare S.p.A., Saronno, VA, Italy), 3 times/day for 30 days, and she was administered nonsteroidal anti-inflammatory drugs (NSAIDs) if needed.

The healing was uneventful and the sutures were removed after 2 weeks.

**Figure 9:**
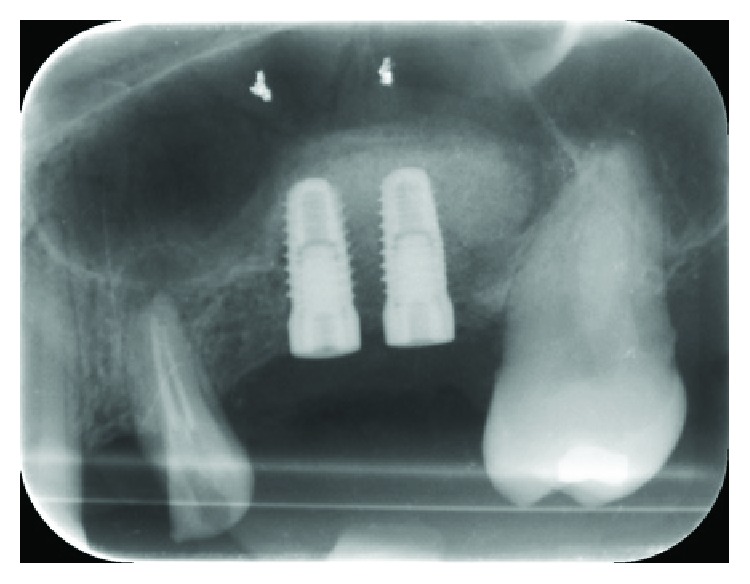
Radiograph taken postimplantation.

At 4 months after the surgery, following a cone-beam CT examination demonstrating a good level of bone regeneration, a mucoperiosteal flap was elevated and two bone tissue carrots 3 mm in diameter were harvested from the implant sites using a trephine bur. Two 3.8 × 9 mm implants were installed (Camlog® Promote® Plus, Camlog Biotechnologies AG, Basel, Switzerland) according to the standard protocol [36]. The insertion torque was 25 N/cm. The mucosal flap covered the fixtures during the healing phase, and the sutures were removed after 10 days. No adverse events occurred (Figure 9).

**Figure 10:**
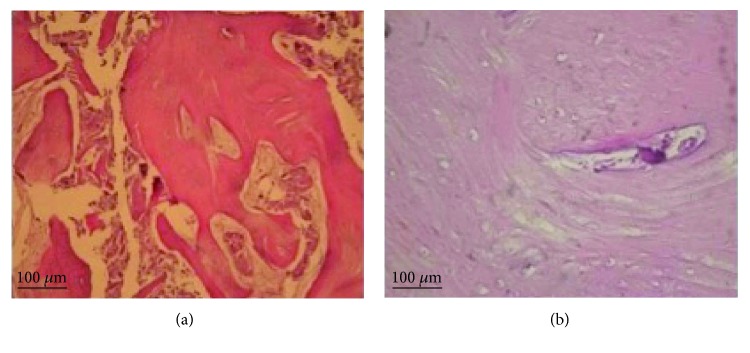
Hematoxylin/eosin staining of samples at 4 months (a, b) after grafting with the Rigenera system. (a) 10x magnification, (b) 40x magnification.

The collected tissues were fixed in a 10% formalin solution and then prepared for microscopic observation in order to determine the ossification grade (Figure 10). Paraffin-embedded tissue sections were cut into 5 *μ*m-thick slices, following which the paraffin slices were immersed in xylene and then in decreasing grades of ethanol (100% to 75%) and deionized water for deparaffinizing and rehydrating the sections. Subsequently, the slide sections were stained with hematoxylin for 1-2 minutes and rinsed in cold water to remove excess stain. The sections were then stained with eosin for 4-5 minutes and rinsed under running tap water. The tissue sections were then immersed in increasing grades of ethanol (from 50% to 100%), and finally after an immersion in xylene, they were coverslipped with a mounting medium. Histological analyses demonstrated that the combination of micrografts with the PLGA scaffold allowed the ossification process. In fact, at 40x magnification (Figure 10(b)), lamellar bone formation was observed, as seen by the presence of a typical Haversian system with the deposition of a calcified matrix.

8 weeks after the implant installation, the following standard prosthetic procedures were performed: implant impression, abutment and structure proof, and cemented prosthesis delivery.

**Figure 11:**
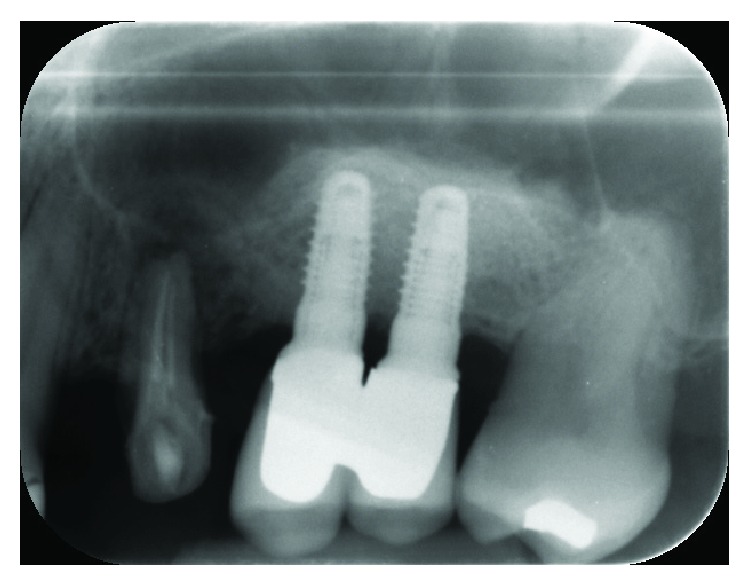
Intraoral radiograph taken after 3 years.

After three years, during the follow-up visit [37], radiographs were taken, which demonstrated an excellent stability of the graft and of the regenerated bone and the success of the rehabilitation (Figure 11).

## 3. Discussion

Usually, a bone graft is the first therapeutic option in cases where the amount of bone is inadequate for implant installation. Autologous bone is considered the gold standard in sinus augmentation procedures but exposes to donor site morbidity. With the Rigenera protocol, the amount of tissue harvested is very little and the donor site is the surgical site itself, thus minimizing the risk of morbidity. In the presented clinical case, healing was uneventful and no sign of tissue harvesting resituated in the palatal flap. The authors did not observe any differences in postoperative soft tissue healing and patient morbidity with respect to the standard procedure due to soft tissue harvesting from the palatal flap. Synthetic materials exhibit a good capability to regenerate an adequate amount of bone, but they do not exhibit the osteoinductive and osteogenic properties needed for bone regeneration. Furthermore, some of these materials show a lack of resorbability even after years from the time of production. This is the reason why the field of bone tissue engineering has focused on techniques such as the use of mesenchymal stem cells [38]. Although many reports suggest that stem cell-based tissue engineering is beneficial, general critiques of cell therapy approaches have included the lack of characterization of the cellular component of the graft [27]. Previous evaluation of micrografts produced by the Rigenera protocol indicated that these cells are positive to mesenchymal cell line markers and negative to hematopoietic and macrophage markers. In fact, cell characterization performed by FACS was positive for several mesenchymal cell markers, including CD90, CD105, and CD73, and negative for CD45 and CD14. Moreover, the Rigenera protocol demonstrated to be able to produce in a few minutes (about 2 min) a cell suspension containing millions of viable cells with a cutoff of 50 *μ*m, opportunely selected by filtration [35]. The behavior of these cells is not clearly known yet, and it could present some risks. However, many studies regarding this topic are being conducted and some researchers have also proposed to use patients' mesenchymal cell micrograft directly, so that the patient is the donor and the receiver at the same time.

A licit criticism related to the use of MSC in a therapeutic procedure is that the graft cell population is composed of nonclonal stromal cells containing stem cells, progenitor cells, and differentiated mesodermal cells, including fibroblasts, and that the advantages connected to their use are more related to their important role in modulating inflammation compared to any stem cell activity [39].

It was demonstrated that micrografts obtained by the Rigenera protocol are able to maintain the osteogenic and regenerative properties because of the content of the progenitor cells [35, 38]. In fact, histological analysis also suggested that the Rigenera protocol facilitates ossification process in the surgical site. In Figure 10, hematoxylin/eosin staining showed the formation of a new bone at 4 months after the maxillary sinus lift procedure, suggesting that the combination of the appropriate biomaterials and the micrografts accelerated the bone-healing process. In particular, the histological analysis showed the presence of bone lamellae, which are concentric rings of bone, surrounding a central channel, or the Haversian canal, containing nerves, blood vessels, and lymph (Figures 10(a) and 10(b)). These lamellae are produced by osteoblasts that secrete extracellular bone matrix with collagen fibers and inorganic phosphate.

The sinus lift surgery performed in this case was associated with a resorbable scaffold HA enriched with progenitor cell micrograft, which was harvested from the palatal periosteum. In particular, the small tissue sample was derived directly from the surgery flap, so the biologic cost was very low.

The scaffold is important to provide the stability and mechanical resistance required to maintain the viability of the cells. Different types of osteoconductive materials could be used as scaffolds. In this case, we chose the PLGA HA-enriched scaffold, which was completely resorbable, as PLGA without HA, but also offered more stability to the graft because of the presence of hydroxyapatite. The micrografts, compared with the other bone grafts, are effective in the regeneration of bone required for implant surgery and are capable of supporting long-term prosthetical load [40, 41].

## 4. Conclusion

This case illustrates that the use of autologous micrografts, which are rich in progenitor cells, in the sinus floor lift procedure is effective in regenerating an adequate amount of bone tissue, with both excellent implant stability and minimum biological sacrifice.

## Abbreviations

PLGA: Poly lactic-co-glycolic acid
NSAID: Nonsteroidal anti-inflammatory drugs
ASA: The American Society of Anesthesiologists.
